# Hybrid FIB milling strategy for the fabrication of plasmonic nanostructures on semiconductor substrates

**DOI:** 10.1186/1556-276X-6-572

**Published:** 2011-10-31

**Authors:** Joshua F Einsle, Jean-Sebastien Bouillard, Wayne Dickson, Anatoly V Zayats

**Affiliations:** 1 Centre for Nanostructured Media, IRCEP, The Queen's University of Belfast, Belfast, BT7 1NN, UK; 2 Department of Physics, King's College London, Strand, London WC2R 2LS, UK

## Abstract

The optical properties of plasmonic semiconductor devices fabricated by focused ion beam (FIB) milling deteriorate because of the amorphisation of the semiconductor substrate. This study explores the effects of combining traditional 30 kV FIB milling with 5 kV FIB patterning to minimise the semiconductor damage and at the same time maintain high spatial resolution. The use of reduced acceleration voltages is shown to reduce the damage from higher energy ions on the example of fabrication of plasmonic crystals on semiconductor substrates leading to 7-fold increase in transmission. This effect is important for focused-ion beam fabrication of plasmonic structures integrated with photodetectors, light-emitting diodes and semiconductor lasers.

## Introduction

Plasmonic nanostructures are finding ever increasing number of applications in various areas of photonics and optoelectronics [1-3]. While initial investigations into the optical properties of plasmonic systems have been almost exclusively done with metallic nanostructures on 'passive' dielectric substrates, such as silica or quartz, the real-world applications in many cases require the use of semiconductor substrates. Recently, there has been a demand on incorporating plasmonic nanostructures in active photonic devices, such as light-emitting diodes (LEDs), semiconductor lasers and photodetectors, to improve their performance [4-7].

For applications in visible and near-infrared spectral ranges, the plasmonic structures need to be fabricated with a precision on the order of tens of nanometers. Conventional microelectronics fabrication methods, such as visible and UV lithography and broad-beam ion etch, do not allow controlling feature sizes on such length scales. The two main methods for the fabrication of plasmonic nanostructures relies on using charged particle beams to structure the material. For example, electron beam lithography can be combined with either lift-off or an etch step to produce nanoscale structures. Electron beam lithography though is not the most efficient process and requires further processing before the final device is created. While robust, this process does not offer sufficient flexibility for quick and rapid prototyping. On the other hand, focused ion beam (FIB) milling is widely accepted as a method of choice for rapid prototyping of electronic and photonic components requiring critical parameters at the subwavelength scale. FIB can sputter away bulk material with nanoscale spatial localisation. The FIB approach offers a simple method to structuring bulk materials, by providing a maskless process that circumvents the pitfalls of resist-based lithography processes. A large variety of photonic and plasmonic devices with structurally controlled optical properties can be created using FIB milling [1-3,8-10]. While excellent for fabrication of plasmonic structures on dielectric substrates, FIB patterning results in the deterioration of optical properties of semiconductors because of ion-beam-induced amorphisation and Ga^+ ^implantation [11-13]. From FIB applications for milling semiconductor materials for transmission electron microscope (TEM) investigations, it is known that the 30 kv FIB damages approximately 50 nm of the GaP crystal through amorphisation (see, e.g., [10,12] and Peterson and Blackwood (2010, personal communication)).

**Figure 1:**
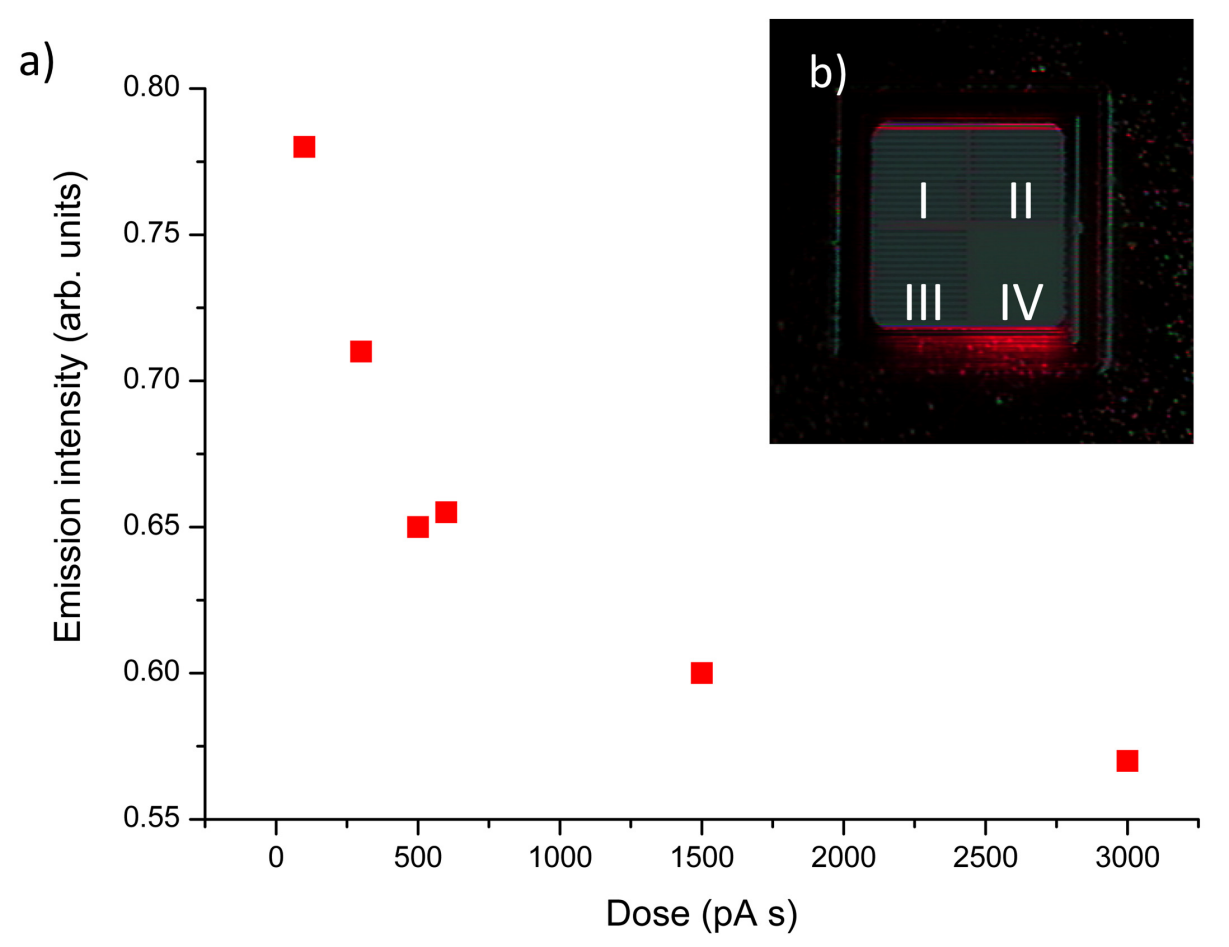
**Optical Performance of FIB patterned LED**. **(a) **LED emission intensity as a function of the FIB exposure dose. **(b) **Optical image of the LED patterned with box mills of different 30 kV FIB doses. LEDs used for milling have been provided by OSRAM Opto-Semiconductors.

The influence of FIB milling on the optical properties of the semiconductor surface can be seen from the performance of two LEDs whose emission face has been etched under different FIB patterning parameters (Figure 1). One LED was patterned using a 30 pA at 30 kV beam setting, while the other LED was etched using 50 pA at 30 kV. The total mill time varied from 0 to 60s. A remarkable degradation in the light emitting from the devices has been observed. Since FIB-milled plasmonic crystals are routinely used now for investigations towards the improvement of LED and photodetector performance, the above-described effect may be very significant in determining a final performance of devices. In this letter, we demonstrate a strategy for minimising FIB-induced effects on plasmonic crystal transmission on semiconductor surfaces. It is known that the substrate damage can be reduced with the use of low energy ions but this also results in a loss of resolution. Here, we employ a hybrid approach to plasmonic structure fabrication to mitigate substrate damage and at the same time maintain high spatial resolution.

## Methods

GaP substrates were used in these experiments as a representative for the InGaAs family of semiconductors. The fabricated plasmonic nanostructures are plasmonic crystals consisting of arrays of periodically arranged cylindrical apertures in a 100-nm Au film deposited on GaP substrates. The Au films were magnetron sputtered on the GaP substrates. Then, an FEI Nova 600 Dual Beam equipped with a Sidewinder FIB column was used to etch away selected regions of the Au film. To precisely control FIB mills, stream patterning files were used. Using FEI's PS Convert, pattern files are generated by inputting FIB-milling parameters such as horizontal field width, spot size, beam overlap (space between points in the pattern) and dwell time. The software then generates a file specifying pixel location and dwell for each point in the pattern. The choice of the input parameters allows controlling the overall depth of the aperture arrays created. Stream files provide fast and easy control over the various patterns required to mill arrays with various accelerating voltage conditions.

**Figure 2:**
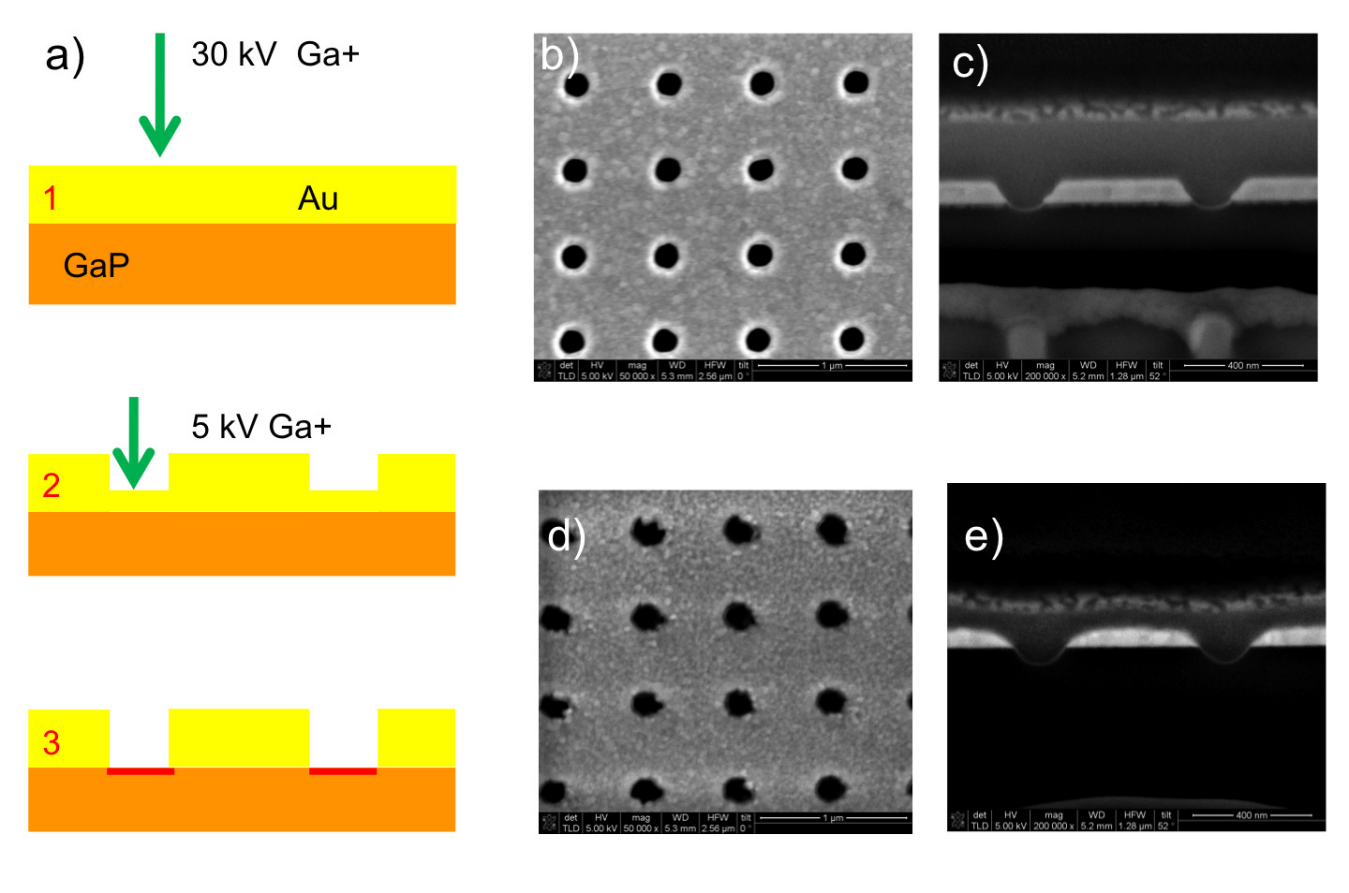
**SPPC hybrid milling routine**. **(a) **Schematic of the hybrid milling routine employed. **(b,d) **SEM images and **(c,e) **SEM cross sections of SPPC crystals in structure A **(b,c) **and structure E **(d,e)**.

The standard approach for the fabrication of surface plasmon polaritonic crystals (SPPC) relies on removing selected regions of Au using ions accelerated through a 30-kV potential. This results in an FIB capable of providing high-resolution patterning capabilities but the high energy ions introduce damage to a semiconductor substrate. A hybrid milling strategy combining high and low energy ion beams has been developed previously for TEM lamella fabrication. It was used however to remove bulk regions of material and not to create fine structures. In this study, we have adopted fabrication which includes initial milling employing focused ions accelerated at 30 kV with a 5-kV ion beam performing the final thinning and polishing to achieve the required thickness and at the same time remove the 30 kV beam-induced damage layer [13]. The use of higher energy ions for nanostructuring allows the process to maintain a high throughput along with high spatial resolution. At the same time, 5 kV ions do not create a deep layer of amorphous material but have disadvantage of low mill rates and decreased resolution. The structures presented below have been created by milling up to 70% of bulk Au film with the standard (30 keV) ion beam energy, and then removing the remaining Au film with the 5 keV ion beam (Figure 2a). While the latter provides lower resolution, the feature size is determined by the high energy ions pre-patterned structure.

For milling optimisation, several plasmonic crystals consisting of square arrays (600 nm period) of cylindrical apertures (200 nm diameter) have been fabricated under different milling conditions (Table 1). Structure A represents the conventional milling approach exclusively using a 30-kV ion beam and not imaging the substrate with the FIB before or after milling the array.

**Table 1:** Hybrid milling conditions used for SPPC fabrication

Structure	30-kV mill time (s)	5-kV mill time (s)
A	5	N/A
B	5	Image only
C	3	22
D	3	45
E	4	45

Structure B was fabricated to investigate the effect of imaging a 30-kV fabricated array with the 5-kV beam. Owing to the reduced signal in the low kV image, perfect overlay of the 5- and -30 kV patterns required in the hybrid FIB approach is challenging to achieve. As a result, the overlay alignment could not be achieved without taking a sequence of high resolution images with the 5 kV ion beam. These were used to bring the 30 kV structure into the field of view such that the 5 kV mill pattern could be accurately overlaid with the 30 kV milled features. The alignment images have the net effect of removing material from the entire region imaged. The amount of gold sputtered was measured via cross-sectional images to be around 10 nm. It would be ideal to be able to eliminate the two images, however the damage induced by taking these two images seems to be minimal as demonstrated in the results shown below.

Structures C and D represent removing 50 nm of Au using the 30 kV beam. The remaining 50 nm of Au in the aperture holes are removed via a combination of imaging and patterning. Finally, for structure E, 70 nm of Au was removed via 30 kV patterning leaving the remainder to be removed with the 5 kV beam. The fabricated structures show good quality of fabricated SPPCs (Figure 2b,c). The cross sections of the structures fabricated by different milling approaches are very similar.

## Results and discussion

**Figure 3:**
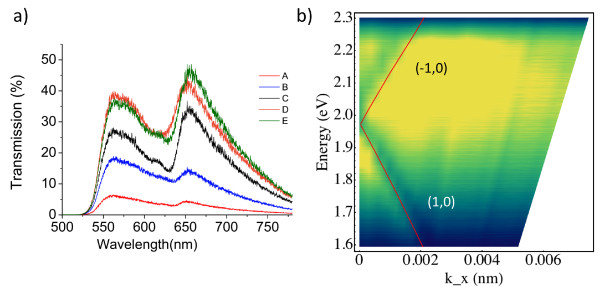
**Hybrid milling optical characterisation**. **(a) **Zero-order transmission spectra of the SPPCs in structures A-E. **(b) **Transmission dispersion measured for SPPCs fabricated using optimum hybrid FIB milling strategy (Structure E). Lines represent the estimation of SPPC band gap using the Bragg conditions.

Optical characterisation of the structures was performed by measuring optical transmission spectra of the plasmonic crystals as described by Bouillard et al. [14]. Both zero-order transmission and transmission dispersion were measured (Figure 3). The observed spectra are typical for plasmonic crystals on high-refractive index substrates [9]. No transmission is observed below about 520 nm where the GaP substrate becomes strongly absorbing. The plasmonic crystal transmission is simpler on high-refractive index substrates than on glass because of the fact that GaP/Au interface does not support surface plasmon polaritons (SPPs) in the spectral range below about 620 nm because of the *ε_GaP _+ *ε*_Au _*> 0, so that only SPP modes on the Au/air interface are important. Two main peaks observed in all structures are associated with (±1, 0) Bloch modes of the Au-Air interface (Figure 3b) that can be derived from the conventional SPP Bragg scattering conditions [3].

All five structures A-E exhibit similar transmission spectra with the same position of the plasmonic resonances, it can be concluded that all single high-energy and double high/low-energy mills have produced aperture arrays with similar parameters and not significantly altered the geometry of the apertures, since the transmission spectra are very sensitive to the shape of the apertures. The most prominent difference in the transmission of the structures is the significantly increased transmission for the structures milled with the low kV approach. The main transmission peak around 660 nm shows a greater than sevenfold increase for the structures made with the hybrid milling when compared to the 30 kV patterning. The standard 30-kV milled structure shows lowest transmission. As seen in device B, simply imaging the structure with low kV ions improves the transmission because of the partial removal of the semiconductor damaged layer. The three structures milled with hybrid approach exhibit highest transmission.

## Conclusion

We have described a hybrid milling approach for fabricating plasmonic crystals on semiconductor substrates. Combining two different accelerating voltages to etch the plasmonic crystal, we can achieve minimal overall damage to the semiconductor substrate keeping high-resolution capabilities of the ion-beam-based techniques. Reducing the amorphisation of the substrate results in over sevenfold increase of the optical transmission of semiconductor/metal nanostructures because of the reduction of the semiconductor surface damage. This FIB-milling process extends the ability of the technology to fabricate plasmonic and other nanostructures on substrates which are usually damaged through traditional FIB patterning approach by maintaining high spatial resolution of high-energy milling and lower damage introducing by low-energy ion beams.

## Abbreviations

FIB: focused ion beam; LED: light emitting diode; SEM: scanning electron microscope; SPP: surface plasmon polarition; SPPC: surface plasmon polaritonic crystal; TEM: transmission electron microscope.

## Competing interests

The authors declare that they have no competing interests.

## Authors' contributions

JFE developed the hybrid milling routine. JFE, JSB and WD carried out optical characterisation measurements. All authors analysed the data. All work was supervised by AVZ. All authors read and approved the final manuscript.
